# Cryptic female choice within individual males – A neglected component of the postmating sexual selection?

**DOI:** 10.1111/jeb.14081

**Published:** 2022-08-21

**Authors:** Jukka Kekäläinen

**Affiliations:** ^1^ Department of Environmental and Biological Sciences University of Eastern Finland Joensuu Finland

**Keywords:** cryptic female choice, egg, fertilization, gamete, haploid selection, monogamy, polygamy, sexual selection, sperm

## Abstract

Cryptic female choice (CFC) is commonly assumed to act only in polyandrous mating systems, which allows females to bias fertilization towards the sperm of particular males. However, accumulated evidence has demonstrated that sperm show significant phenotypic and genotypic variation also within single ejaculates, which have important consequences for offspring phenotype and fitness. Here, I argue that these neglected sources of intra‐male sperm variation often allow CFC to act also within individual males and facilitate fertilization bias towards genetically compatible (or otherwise preferred) sperm haplotypes. In this article, I explain prerequisites for within‐male CFC, the criteria for demonstrating it and summarize accumulated evidence for this emerging selection process. Then, I evaluate prevalence of within‐male CFC and review its potential evolutionary consequences. The aim of this article is to broaden the current definition of CFC by demonstrating that CFC has potential to act in all mating systems, in both internally and externally fertilizing species. Incorporation of the within‐male CFC concept into the current models of sexual selection may provide novel insights into the deeper understanding of selective factors driving the evolution of mating systems and reproductive proteins. Finally, within‐male CFC towards particular sperm haplotypes may increase our understanding of non‐Mendelian inheritance.

## Graphical abstract

Sperm cells show significant phenotypic and genotypic variation within single ejaculates. This variation can enable cryptic female choice to act also within single males (their ejaculates) and bias fertilization towards genetically compatible or otherwise favourable sperm cells.

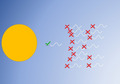

## 1 INTRODUCTION

In many species, sexual selection continues after the mating in the form of sperm competition (intrasexual selection), where ejaculates from different males compete to fertilize females' eggs, and cryptic female choice (intersexual selection), where females bias paternity towards particular males over others (Birkhead & Pizzari, 2002). Both of these sexual selection episodes require that female mate with multiple males (polyandry). In other words, ejaculates of several males should either be simultaneously present during the fertilization event or there must at least be a risk that female will mate with more than one male prior to fertilization (Dougherty et al., 2016; Firman et al., 2017).

Cryptic female choice (CFC) has originally been suggested to act via more than 20 different mechanisms, varying from the female copulatory behaviour to postbirth investment in offspring (Eberhard, 1996). However, most of the earlier studies have investigated mechanisms that occur just after the mating, but prior (or during) the fertilization. Accumulating evidence suggests that CFC often acts via prefertilization cellular‐ and molecular‐level processes that facilitate mate choice at the level of the gametes (gamete‐mediated mate choice, GMMC: Kekäläinen & Evans, 2018). Given that these processes potentially allow females to execute CFC even at the level of individual sperm cells, GMMC potentially enables much more accurate mate choice than any other mechanism of CFC or premating sexual selection. Furthermore, since some form of biochemical communication between male and female gametes most likely occur in all sexually reproducing species, GMMC can act even in species where premating sexual selection is not possible.

CFC (and GMMC) can result either in (a) *directional* selection, when females share postmating preferences on certain (‘high quality’) male phenotypes, or (b) *nondirectional* selection, when preference criteria will differ across male–female combinations (Firman et al., 2017; Kekäläinen & Evans, 2018). Most of the available evidence support nondirectional selection hypothesis and especially genetic incompatibility, inbreeding or hybridization avoidance seem to act as important mediators in the process (Kekäläinen & Evans, 2018). However, the adaptive significance of CFC has remained largely unclear, since it has been demonstrated that cryptic female choice and polyandry often do not provide clear direct or indirect (genetic) benefits for females (e.g. Firman et al., 2017; Slatyer et al., 2012; Sutter et al., 2019). Therefore, is it possible that CFC is also driven by fitness benefits that are not directly dependent on polyandry? Here, I argue that GMMC also operate within each individual ejaculate, and bias fertilization towards specific sperm haploid genotypes. I also envisage that benefits gained from such ‘within‐male cryptic female choice’ (hereafter ‘within‐male CFC’) may often outweigh the benefits of CFC acting at the level of the male individuals. In the following sections of this paper, I first define the conceptual background and prerequisites for within‐male CFC and draw together existing evidence for the phenomenon. Then, I describe methodological approaches how to investigate within‐male CFC, evaluate its prevalence and finally, emphasize its potential evolutionary significance.

## 2 SPERM COMPETITION AND CRYPTIC FEMALE CHOICE WITHIN SINGLE MALES (EJACULATES)

In most species, males produce many more sperm cells than what would be necessary to fertilize all the available female's eggs, which result in competition between sibling sperm within each ejaculate (hereafter ‘within‐male sperm competition’: Sutter & Immler, 2020). Importantly, although the risk and intensity of sperm competition typically vary significantly across species, individual males and mating events, within‐male sperm competition is expected to occur during all the fertilization events. Additionally, within‐male sperm competition occurs both in polygamous and in monogamous mating systems and is independent of the fertilization mode (internal vs. external) (Ezawa & Innan, 2013). Importantly, Ezawa and Innan (2013) also demonstrated that competition among sibling sperm can often be much more intense than sperm competition occurring between individual males.

Similarly, in many species, females allow only a minor subset of ejaculated sperm to swim in the vicinity of the unfertilized eggs. For example, in humans, up to hundreds of millions of sperm cells are released during sexual intercourse, but the female reproductive tract enables only a strictly selected sperm subpopulation to proceed in the fertilization site, the ampullary segment of the fallopian tube (Hanevik et al., 2016; Holt & Fazeli, 2015). Accordingly, only about one of 1 000 000 sperm can enter the fallopian tubes, and only a few of these cells eventually reach the unfertilized oocyte (Eisenbach & Giojalas, 2006). Therefore, along with within‐male sperm competition, also female‐induced sperm selection can be extremely fierce. Traditionally, it has been thought that the primary function of this sperm selection process is to eliminate fertilization‐incompetent or poor‐quality sperm (e.g. Pitnick, Wolfner, & Suarez, 2009). However, accumulating evidence has suggested that these sperm selection mechanisms have an additional role in mediating GMMC towards genetically compatible (or otherwise suitable) reproductive partners (Holt & Fazeli, 2015; Jokiniemi, Kuusipalo, et al., 2020; Jokiniemi, Magris, et al., 2020). Importantly, given that all the individual gametes are genetically unique, it can be expected that different sperm cells differ in their compatibility with the female's eggs. This raises the possibility that GMMC (and thus CFC) may have evolved to ‘discriminate’ compatible sperm genotypes also within each male's ejaculate.

## 3 PREREQUISITES FOR WITHIN‐MALE CRYPTIC FEMALE CHOICE

Within‐male CFC can be expected to evolve, if: (1) individual sperm cells exhibit phenotypic variation; (2) sperm phenotypic variation is heritable; and (3) sperm phenotypes affect offspring phenotype and/or fitness. Although sperm phenotypic variation (first criteria) has been widely demonstrated (e.g. Higginson & Pitnick, 2011; Pitnick, Hosken, & Birkhead, 2009; Stewart et al., 2016), it has commonly been assumed that all the fertile (‘healthy’) sperm are equivalent of siring viable offspring (Alavioon et al., 2017). This assumption is based on the findings that during spermatogenesis, developing spermatids effectively share their cytoplasm through cytoplasmic bridges, which has been thought to homogenize their gene products (RNA and proteins), when individual sperm cells should be incapable of expressing their haploid genotypes. Such homogenization is expected to be beneficial for males (i.e. diploid organisms) as it reduces the functional differences and thus within‐ejaculate competition among sperm cells, and this way reduces the risk of a conflict between diploid males and their haploid gametes (Immler, 2019; Nadeau, 2017). Given that this conflict can significantly reduce the competitive ability of the male ejaculate as a whole, males can improve their sperm competition success against other males by minimizing gene and protein expression differences between their gametes. However, despite this benefit, recent findings have indicated that a number of sperm genes are expressed during the haploid stages of spermatogenesis and that products of these genes are often incompletely shared between developing spermatozoa (Bhutani et al., 2021; Nadeau, 2017). Furthermore, it has been demonstrated that sperm phenotypes (such as swimming behaviour) are associated both with their haploid genotypes (second criteria, Alavioon et al., 2017; Borowsky et al., 2019) and sperm mRNA (gene transcript) profile (Lambard et al., 2004). Together this evidence indirectly indicates that haploid selection within ejaculates may be far more common than we have previously assumed.

Some recently published evidence also suggests that sperm phenotypic variation can have important consequences for the offspring phenotype and fitness (third criterion). For example, Rathje et al. (2019) demonstrated in mice that sperm motility and morphology is dependent on the type of the sex chromosome (X vs. Y) sperm cells carry, leading to sex ratio skew in offspring. Furthermore, Alavioon et al. (2017) demonstrated in zebrafish (*Danio rerio*) that selection at the level of the haploid spermatozoa can affect offspring survival and reproductive success. Subsequently, Alavioon et al. (2019) demonstrated that within‐ejaculate sperm selection can also modify offspring health and reproductive ageing. Finally, earlier studies have shown that within‐ejaculate differences in sperm longevity can have important consequences for offspring survival and development (Crean et al., 2012; Immler et al., 2014). Although these latter studies did not investigate haploid selection directly, both studies indicate that sperm phenotypic differences can be associated with offspring phenotypes. However, more studies are needed to better understand the exact physiological and molecular mechanisms behind sperm phenotypic variation and to find out how such variation shape offspring fitness.

## 4 EVIDENCE FOR WITHIN‐MALE CRYPTIC FEMALE CHOICE

Even though CFC has been demonstrated to occur in number of externally and internally fertilizing species (e.g. Alonzo et al., 2016; Fitzpatrick et al., 2020; Gasparini & Pilastro, 2011; Løvlie et al., 2013; Lymbery et al., 2017; Rosengrave et al., 2016), both its mechanistic basis and adaptive significance have remained elusive. CFC is often mediated via cellular and molecular‐level interactions (GMMC, see above), which in internally fertilizing species are occurring ‘hidden’ within the female reproductive tract and are thus technically challenging to investigate (Firman et al., 2017). Demonstrating within‐male CFC is even more challenging as it additionally requires investigating interactions between individual gametes. I argue that this analytical challenge is the primary reason why experimental evidence for within‐male CFC has remained scarce.

The idea that female‐induced sperm selection could act also within ejaculates is not new, and for example, Birkhead et al. (1993) has proposed that females may have evolved to exercise sperm selection both at the level of individual males and within their ejaculates. This theoretical study, along with some experimental evidence indicates that within‐male CFC may be widespread. First, it has been shown that in plants, competition (and thus selection) among pollen from the same individual males can significantly increase the fitness of the seedlings (e.g. Niesenbaum, 1999). Given that haploid gene expression in pollen is well established, this suggest that selection (i.e. CFC) among individual male gametes may be common in plant species (see Arunkumar et al., 2013). Furthermore, as mentioned above, recent animal studies have demonstrated that within‐ejaculate variation in sperm phenotype can have important consequences for offspring performance and development (Alavioon et al., 2017, 2019; Immler et al., 2014). This indicates that potential benefits females could gain from within‐male CFC can be significant, which in turn may have facilitated the evolution of within‐male CFC also in animals. However, so far, only a few studies have shown direct evidence for within‐male CFC.

Nadeau (2017) demonstrated that the combination of gametes at fertilization is not random but can favour selective fusion between specific eggs and sperm haploid genotypes. Accordingly, Nadeau (2017) found 12 genes, which alleles (and thus gametes carrying these alleles) combine non‐randomly at fertilization, leading to transmission ratio distortion (TRD: non‐Mendelian transmission of alleles from parents to their offspring). The most likely explanation for the finding is that eggs (or female reproductive tract in general) may actively bias fertilization towards specific sperm cells based on their haploid genome. Similarly, it has been demonstrated that in three‐spined sticklebacks, eggs can selectively fuse with the sperm that carry complementary major histocompatibility complex (MHC) alleles and this way ensure optimal MHC divergence of the offspring (Lenz et al., 2018). Although detailed molecular‐level mechanisms of the findings remained unclear, it has been suggested that female reproductive tract or eggs can recognize specific sperm surface molecular markers and this way bias the fertilization towards the sperm of particular males (Holt & Fazeli, 2015). It seems likely that these same cell surface markers may play an important role also in sperm selection (CFC) within each individual ejaculate.

Crucially, only a handful of potentially interacting gamete surface compatibility genes have so far been identified (reviewed by Kekäläinen, 2021). Therefore, molecular‐level mechanism of CFC, both at the level of individual males and their haploid sperm, have remained largely unclear (Box 1). However, in principle, all the haploid‐expressed sperm gene products (RNAs and proteins) could act as potential molecular targets of within‐male CFC. Interestingly, it has been shown that RNA transcripts of up to several thousands of sperm genes are not equally shared among developing spermatids (Bhutani et al., 2021; Joseph & Kirkpatrick, 2004). Classic example of these genes is the mouse selfish genetic element, the *t* haplotype (Lindholm et al., 2016). Additionally, it has been envisaged that de novo synthesis of both RNA and proteins may occur also in mature sperm (reviewed by Immler, 2019; Santiago et al., 2022, see also Kekäläinen et al., 2022), which could potentially increase intercellular variation in sperm haploid gene expression even further. Identifying these differentially expressing genes and their functional importance for sperm and offspring phenotypic variation should be the key aim of the future studies.

BOX 1 **Outstanding questions for future studies**

- What are the molecular (gene, RNA and protein) targets of within‐male CFC?
- Do females or their eggs distinguish between haploid sperm genotypes and how it could happen?
- What fitness benefits within‐male CFC offers for females or males?
- Is within‐male CFC more prevalent in monogamous species?
- Is within‐male CFC more intense in internally fertilizing species or species with intense sperm selection?
- What is the relative importance of within‐male and between‐male CFC in the evolution of reproductive proteins and mating systems?

## 5 HOW TO INVESTIGATE AND DEMONSTRATE WITHIN‐MALE CFC?

Genetic compatibility of the sperm and eggs is likely determined by the complex network of genetic interactions (Kekäläinen, 2021). These interactions can occur among the alleles at the same locus (dominance) and between different loci (epistasis). However, I envisage that especially epistasis may play an important role in within‐male CFC. Both favourable (compatible) and unfavourable (incompatible) allele combinations between epistatically interacting genes are expected to show deviation from Mendelian expectations in a given population (i.e. TRD). Detection of such allele pairs is possible, for example by genome‐wide screening of large number of individuals with known family structure, such as parent‐offspring trios and by searching over‐ and under‐representation of specific male and female allele pairs among the offspring (Ackermann & Beyer, 2012). Therefore, within‐male CFC and associated fertilization bias towards genetically compatible sperm haplotypes should be detectable directly from genomic data. However, in addition to within‐male CFC (biased fertilization), TRD can also result from other selection processes occurring during meiosis (meiotic drive), gametogenesis (e.g. reduced gamete production) and embryo development (selective offspring mortality) (Fishman & McIntosh, 2019; Nadeau, 2017). Therefore, identification of allelic incompatibilities from the genome‐wide data only is not sufficient to demonstrate that within‐male CFC has occurred but can still reveal potential candidate genes for future experimental studies (see Kekäläinen, 2021).

Since within‐male CFC acts prior or during the fertilization process, experimental demonstration of within‐male CFC requires identification of individual sperm cells that eventually fertilize the available oocytes. Generally, this could be performed, for example by comparing genetic composition and/or RNA transcript profiles of the unfertilized and fertilized oocytes in relation to available sperm genotypes (or their RNA content). Genetic screening of the gametes can be based on certain candidate genes, such as MHC (Lenz et al., 2018), but rapid development of next‐generation sequencing techniques (such as pooled whole genome sequencing) has also opened new opportunities to identify genome‐wide patterns of allelic transmission biases and underlying mechanisms of TRD (Fishman & McIntosh, 2019). Similarly, modern single‐cell transcriptomics techniques now allow simultaneous measurements of mRNA concentration from tens of thousands of individual gametes (e.g. Tomoiaga et al., 2020). This opens up unique possibilities for investigating within‐male CFC towards haploid‐expressed sperm genes.

To confirm that allelic transmission bias results from within‐male CFC and not from the incompatibility avoidance mechanisms at the diploid genotype level, it is important to ensure that observed genotype bias is independent of zygote and embryo mortality. This can most readily be confirmed in externally fertilizing model species, where sperm selection and fertilization trials can be performed in vitro and where embryo development and mortality can be easily observed (see Lenz et al., 2018). In internally fertilizing taxa such demonstration requires controlled breeding designs, combined with a good prior knowledge of parental genotypes (Arends et al., 2022; Fishman & McIntosh, 2019; Nadeau, 2017) or in vitro experiments, where sperm haploid selection prior to fertilization is investigated in the presence of female reproductive tract secretions (reviewed by Kekäläinen & Evans, 2018), such as follicular fluid or cervical mucus in humans (Fitzpatrick et al., 2020; Jokiniemi, Kuusipalo, et al., 2020; Jokiniemi, Magris, et al., 2020).

## 6 PREVALENCE OF WITHIN‐MALE CFC

Evidence that CFC (or polyandry in general) would be beneficial for females (or males) is surprisingly weak (Firman et al., 2017; Sutter et al., 2019). I argue that one potential explanation for this finding is the fact that earlier studies have almost exclusively assumed that females can gain benefits from CFC only when mating with multiple males. However, sperm of each individual males on average share only 50% (range 0–100%) of their genes (Immler & Otto, 2018). Therefore, in extreme cases, within‐ejaculate relatedness among individual sperm cells can be even lower than the genetic divergence between unrelated males. It has also been demonstrated that different sperm subpopulations are genetically distinct (Alavioon et al., 2017; Borowsky et al., 2018) and show differences in RNA and protein composition (Martínez‐Pastor, 2021). Together, all these facts raise the possibility that females can gain significant fitness benefits from their choosiness also in monogamous matings. In the light of this evidence, I argue that one and possibly the most important explanation for difficulties to demonstrate the benefits of polyandry is the fact that CFC (and sperm competition) provides females significant benefits also when acting within individual males. In other words, the relative fitness advantage females generally gain from polyandrous mating in relation to monandry may be smaller than we have assumed.

As explained above, in many species, only a tiny fraction of the phenotypically and genetically different sperm cells end up fertilizing the eggs, which creates opportunities for CFC to act among these genetically unique cells and/or sperm subpopulations. Given that along with sperm, individual eggs also differ genetically, it can be expected that different sperm–egg‐combinations differ in their genetic ‘quality’ or compatibility, which in turn can lead to fertilization bias (nonrandom fertilization) towards genetically compatible (or otherwise preferred) sperm haplotypes. Such selection process can be expected to occur in most sexually reproducing species, irrespective of the type of the mating system or fertilization mode (Box 1). However, it has been demonstrated that intensive between‐male sperm competition (i.e. polyandry) selects for suppressing sperm haploid gene expression (Ramm et al., 2014), indicating that within‐male CFC may be particularly common (and more intense) in monogamous species. Similarly, I envisage that intensity of within‐male CFC should be especially high in mammals (and possibly in other internally fertilizing species), where only a tiny proportion of ejaculated sperm cells are able to proceed in the vicinity of unfertilized eggs (see e.g. Eisenbach & Giojalas, 2006). Furthermore, since sperm haploid genotypes have been demonstrated to affect sperm swimming behaviour, but not sperm length (Borowsky et al. (2019), see also Morrow & Gage, 2001), it seems likely that within‐male CFC targets more strongly on various sperm behavioural traits than sperm morphology. Finally, within‐male CFC should be absent (or weak) in haplodiploid species, where all the sperm are genetically identical, and sperm phenotypic variation thus is not heritable.

## 7 EVOLUTIONARY SIGNIFICANCE OF WITHIN‐MALE CFC

Besides broadening the current definition of CFC, within‐male CFC may increase our understanding on the evolution of reproductive proteins and mating systems (Box 1). First, it has been demonstrated that genes coding gamete surface proteins are among the fastest evolving genes known and sexual selection is widely believed to act as a key driver of this evolution (Springate & Frasier, 2017; Swanson & Vacquier, 2002). However, evolutionary rate of reproductive proteins does not necessarily correlate with the intensity of sexual selection as divergence rate of reproductive proteins is often elevated also in monogamous species (e.g. Ezawa & Innan, 2013; Ramm et al., 2008). Ezawa and Innan (2013) suggested that this seemingly counterintuitive finding can be explained by within‐male sperm competition. However, authors also argued that the within‐male sperm competition alone cannot explain long‐term divergence of reproductive proteins, which additionally requires continual coevolution between male and female reproductive proteins. This in turn raises the possibility that within‐male CFC may act as an important, but currently neglected selective force in reproductive protein evolution, especially among sperm genes that are expressing in the haploid phase.

Second, postmating sexual selection is generally expected to be weak in monogamous mating systems (Kvarnemo, 2018), which in turn would mean that CFC should not play an important (if any) role in such species. Furthermore, different male–female combinations often differ in their genetic compatibility, which has been assumed to lead to the evolution of polyandry. In other words, selection mechanisms for postmating incompatibility avoidance are expected to evolve only in polygamous mating systems (but see Jokiniemi, Kuusipalo, et al., 2020; Jokiniemi, Magris, et al., 2020; Magris et al., 2021). However, these traditional models of sexual selection have almost completely ignored the possibility that both sperm competition and cryptic female choice among ‘sibling’ sperm can occur also in monogamous species (but see Ezawa & Innan, 2013; Sutter & Immler, 2020). Therefore, within‐male CFC may also contribute to the evolution of mating systems.

Finally, one of the most important premises of genetics, Mendel's laws of inheritance, postulate that the union of gametes at fertilization is completely random (Nadeau, 2017). However, probability of fertilization is also strongly dependent on complex network of interacting male and female genes (Kekäläinen, 2021). The vast number of potentially interacting genes poses an enormous analytical challenge to statistically identify functionally important genetic interactions from the genomes due to required corrections for multiple hypothesis testing (e.g. Ackermann & Beyer, 2012). In other words, many important allelic distortions can remain undetected due to the lack of statistical power. Thus, it is possible that non‐Mendelian fusion of the gametes (i.e. TRD) may be far more common than we have assumed and that within‐male CFC may act as an important mediator of the process. Therefore, within‐male CFC can also facilitate our understanding of the mechanisms of inheritance.

## CONFLICT OF INTEREST

The author has no conflict of interests to declare.

### PEER REVIEW

RESPONSE TO PEER REVIEW TRANSPARENCY OPTION (reviewer reports, author responses, and decision letter linked from publons): Agree

For every manuscript submitted on or after 17 September 2019, if the author response included above is “agree”, be sure to do the following: include statement “The peer review history for this article is available at https://publons.com/publon/10.XXXX/XXX.XXXXX” (fill in article DOI) in both the article header on WOL and the PDF. See example at https://doi.org/10.1111/cge.13363.

## Data Availability

This article contain no data.
